# Bilateral Serous Macular Detachment After Attempted Suicide with Pregabalin

**DOI:** 10.4274/tjo.70923

**Published:** 2018-10-31

**Authors:** Burak Tanyıldız, Baran Kandemir, Mehmet Serhat Mangan, Aise Tangılntız, Eren Göktaş, Şaban Şimşek

**Affiliations:** 1 İstanbul Kartal Dr. Lütfi Kırdar Training and Research Hospital, Ophthalmology Clinic, İstanbul, Turkey; 2 İstanbul Okmeydanı Training and Research Hospital, Ophthalmology Clinic, İstanbul, Turkey; 3 Bezmiâlem Vakıf University Faculty of Medicine, Department of Psychiatry, İstanbul, Turkey

**Keywords:** Optical coherence tomography, pregabalin, serous macular detachment, suicide

## Abstract

A 24-year-old female presented with bilateral vision loss following attempted suicide with pregabalin. Her best-corrected visual acuity (BCVA) was 20/40 in the right eye and 20/50 in the left eye. The bilateral visual disturbance was associated with serous macular detachment. Fundus examination of both eyes showed foveal serous retinal detachment, which was confirmed by optical coherence tomography. Topical nepafenac 0.1% eye drops were started as single drop every 8 hours for 4 weeks. One month later, the serous macular detachment had regressed and BCVA increased to 20/20 in both eyes. To the best of our knowledge, this is the first reported case of bilateral serous macular detachment presumably caused by pregabalin intoxication.

## Introduction

Pregabalin is a gamma-aminobutyric acid (GABA) analogue with antiepileptic, analgesic, and anxiolytic effects.^1,2^ These effects occur when pregabalin binds to presynaptic voltage-gated calcium channels to regulate calcium entry into the cell, thereby reducing the release of neurotransmitters such as glutamate, norepinephrine, substance P, and calcitonin gene-related peptide.^3,4^

There has been an increase in publications regarding the abuse of pregabalin in recent years.^5,6^When taken in high doses, pregabalin may result in side effects such as affective disorders, somnolence, confusional state, agitation, and restlessness.^7^

Common ocular side effects of pregabalin include blurred vision^7^ and diplopia.^8^ Less frequent side effects such as ocular pain, photopsia, and irritation have also been reported.^7^

In this case report, we present a patient with bilateral serous macular detachment following attempted suicide with oral pregabalin.

## Case Report

A 24-year-old female patient presented with complaints of blurred vision for 2 weeks. According to the patient’s history, she had attempted suicide 2 weeks earlier by taking 15 tablets of pregabalin (Lyrica, 300 mg; Pfizer, Tadwort; United Kingdom) and was brought to the emergency department of another center with loss of consciousness and seizures. According to the patient’s discharge report, her blood pressure was 100/60 mmHg, heart rate was 165/minute, respiration rate was 34/minute, and body temperature was 36.8 °C in the initial examination done in emergency services. Hemogram and biochemical values were within normal limits. Arterial blood gas analysis done during follow-up in intensive care showed pH: 6.79, PaO_2_: 45 mmHg, PaCO_2_: 55 mmHg, HCO_3_: 7.9 mmol/L, and BE: -33.6 mmol/L. Blood drug level was not analyzed. The patient exhibited deep metabolic acidosis and convulsions and was treated with intravenous hydration, 20 ampules of NaHCO_3 _and 0.05 mg/kg midazolam (Dormicum, Roche). After treatment, arterial blood gas analysis showed pH: 7.41, PaO_2_: 145 mmHg, PaCO_2_: 31.8 mmHg, HCO_3_: 18.8 mmol/L, and BE: -3.3 mmol/L. On day 3 of follow-up, the patient’s general condition was improved and she was conscious and alert. She had developed blurred vision during this time, and was referred to the ophthalmology department upon discharge. Ophthalmologic examination revealed bilateral serous exudative macular detachment, upon which the patient was referred to our clinic for further examination and treatment.

**Figure 1:**
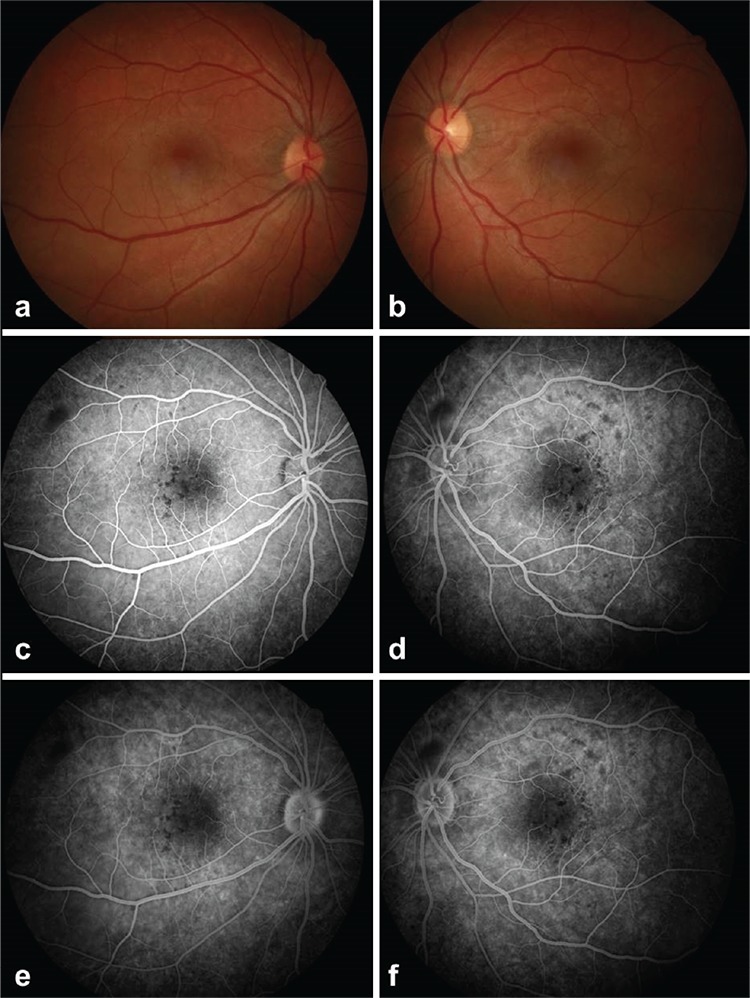
Color fundus photography and fundus fluorescein angiography of a 24-year-old female patient at her initial presentation to our clinic; (a, b) color fundus image shows absent foveal reflex in both eyes due to subretinal fluid; (c, d) fundus fluorescein angiography shows bilateral spots of hypofluorescence starting in early phases and continuing in late phases (e, f) in the posterior pole.

**Figure 2:**
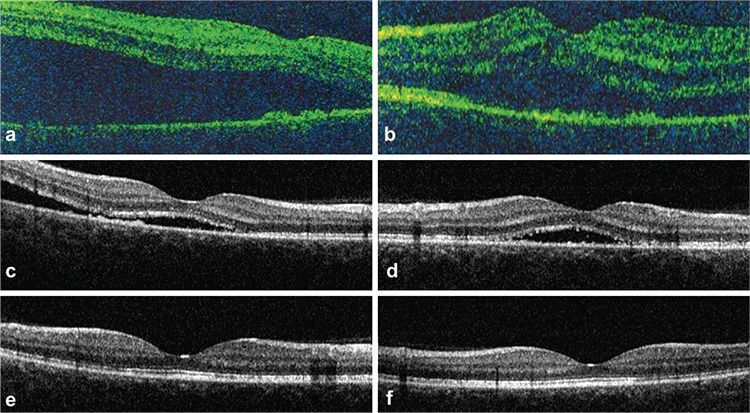
Optical coherence tomography images obtained in another center and in our center after discharge from intensive care; (a, b) optical coherence tomography taken in the other center 3 days after the suicide attempt shows bilateral serous detachment that is more pronounced in the right eye; (c, d) optical coherence tomography taken in our clinic 1 week later shows significant reduction of the subretinal fluid in both eyes compared to the initial images; (e, f) in optical coherence tomography taken 1 month later the subretinal fluid had totally regressed

On examination in our clinic, her best corrected visual acuity (BCVA) was 20/40 in the right eye and 20/50 in the left eyes. Anterior segment examination was normal. Intraocular pressure was within normal limits. Foveal reflex was absent bilaterally on fundoscopic examination (Figures 1a, b). Fundus fluorescein angiography revealed foci of hypofluorescence in the posterior pole starting in the early phases and continuing in the late phases (Figures 1c, d, e, f). Optical coherence tomography (OCT) images obtained in the other center and in our clinic showed subretinal fluid in both eyes (Figures 2a, b, c, d). Based on the patient’s history and examination findings, the serous macular detachment was believed to be a result of pregabalin intoxication. Treatment was started with topical nepafenac 0.1% (Nevanac Alcon, Forth Worth, Texas, United States of America) 3 times a day. The subretinal fluid was totally resolved after 1 month of treatment (Figures 2e, f). Topical treatment was discontinued. On examination 3 months after her initial presentation, BCVA was 20/20 in both eyes and no subretinal fluid was evident on OCT.

## Discussion

Pregabalin is a structural analogue of GABA. It binds to the alpha-2-delta subunits of voltage-gated calcium channels to block calcium influx, resulting in reduced release of excitatory neurotransmitters such as glutamate, norepinephrine, substance P, and calcitonin gene-related peptide.^9^ This mechanism of action led to the use of pregabalin in disorders such as neuropathic pain, epilepsy, and anxiety.^9,10,11^

In the case presented here, a 24-year-old woman developed blurred vision due to serous macular detachment after attempting suicide using pregabalin. Her lack of any relevant medical or family history and the absence of significant systemic pathology other than metabolic acidosis during her stay in intensive care suggest that the serous detachment occurred as a result of the effect of pregabalin. In the literature there is another case reported from Turkey in which unilateral hemorrhagic macular infarct occurred following a suicide attempt using pregabalin, alcohol, and marijuana.^12^ The authors proposed that the macular ischemia in this case developed secondary to marijuana-related arteritis and impaired vascular autoregulation as well as pregabalin-related systemic hypotension. In our case, we suspect the hypofluorescent spots observed in fluorescein angiography and the subretinal fluid observed in OCT may have resulted from a vascular filling defect in the choroidal vessels and increased choroidal vascular permeability which likely developed due to the effect of pregabalin. However, data about the choroidal circulation and thickness were insufficient due to our inability to perform indocyanine green angiography and OCT with enhanced depth imaging.

Pregabalin has a wide range of indications for therapeutic use. It is indicated for patients with peripheral neuropathic pain, fibromyalgia, epilepsy, generalized anxiety disorder, and partial convulsions.^7^ The number of publications reported on the misuse and abuse of pregabalin has increased in recent years.^5,13,14^ Individuals with a history of opioid abuse are particularly prone to abuse pregabalin.^14^ In conclusion, consumption at high doses due to misuse or abuse is possible with pregabalin, which has such a wide range of indications. Although there are limited reports in the literature regarding the potential ocular side effects of pregabalin, a detailed drug use history must be obtained whenever ophthalmologists detect serous macular detachment or macular infarct. Randomized controlled studies are needed in order to better understand the dose-dependent or dose-independent effects of pregabalin on the retina and choroid.
